# Role of Structure and Glycosylation of Adsorbed Protein Films in Biolubrication

**DOI:** 10.1371/journal.pone.0042600

**Published:** 2012-08-15

**Authors:** Deepak H. Veeregowda, Henk J. Busscher, Arjan Vissink, Derk-Jan Jager, Prashant K. Sharma, Henny C. van der Mei

**Affiliations:** 1 Department of Biomedical Engineering, University Medical Center Groningen and University of Groningen, Groningen, The Netherlands; 2 Department of Oral Maxillofacial Surgery, University Medical Center Groningen and University of Groningen, Groningen, The Netherlands; University of South Florida College of Medicine, United States of America

## Abstract

Water forms the basis of lubrication in the human body, but is unable to provide sufficient lubrication without additives. The importance of biolubrication becomes evident upon aging and disease, particularly under conditions that affect secretion or composition of body fluids. Insufficient biolubrication, may impede proper speech, mastication and swallowing, underlie excessive friction and wear of articulating cartilage surfaces in hips and knees, cause vaginal dryness, and result in dry, irritated eyes. Currently, our understanding of biolubrication is insufficient to design effective therapeutics to restore biolubrication. Aim of this study was to establish the role of structure and glycosylation of adsorbed protein films in biolubrication, taking the oral cavity as a model and making use of its dynamics with daily perturbations due to different glandular secretions, speech, drinking and eating, and tooth brushing. Using different surface analytical techniques (a quartz crystal microbalance with dissipation monitoring, colloidal probe atomic force microscopy, contact angle measurements and X-ray photo-electron spectroscopy), we demonstrated that adsorbed salivary conditioning films *in vitro* are more lubricious when their hydrophilicity and degree of glycosylation increase, meanwhile decreasing their structural softness. High-molecular-weight, glycosylated proteins adsorbing in loops and trains, are described as necessary scaffolds impeding removal of water during loading of articulating surfaces. Comparing *in vitro* and *in vivo* water contact angles measured intra-orally, these findings were extrapolated to the *in vivo* situation. Accordingly, lubricating properties of teeth, as perceived in 20 volunteers comprising of equal numbers of male and female subjects, could be related with structural softness and glycosylation of adsorbed protein films on tooth surfaces. Summarizing, biolubrication is due to a combination of structure and glycosylation of adsorbed protein films, providing an important clue to design effective therapeutics to restore biolubrication in patients with insufficient biolubrication.

## Introduction

Water forms the basis of all biological lubrication systems in the human body, but is unable to provide sufficient lubrication without biological lubricant additives, such as hydrophilic sugars [1] and mucins [2]. Insufficient biolubrication can yield severe discomfort, and especially occurs in the elderly and patients with Sjögren's syndrome, a syndrome which includes dryness of the mouth impeding proper speech and mastication [3]–[5], dry, irritated eyes [6], vaginal dryness [7] and excessive friction and wear of articulating cartilage surfaces in hips and knees [8]. Interestingly, many Sjögren's patients suffer from more than one symptom of insufficient biolubrication [9]. Patients suffering from Sjögren's syndrome scored significantly lower on six out of eight points of a commonly applied health related quality of life questionnaire than a general population as well as for the summary scores of that questionnaire for physical and mental functioning [10]. This suggests that insufficient biolubrication constitutes a physical condition affecting the entire body, related to the general absence of secretion of an appropriate biological lubricant.

Often, biological lubricants form an adsorbed, so-called conditioning film that provides low friction with an opposing surface, due to the immobilization of water by adsorbed hydrophilic sugars [1], [11]–[13], or repulsion between polymer-brush bearing surfaces [14]–[16]. Lubricating films in the human body should be able to withstand high pressures, such in articulating cartilage surfaces in the hip or knee [17], while ocular lubricating films function in the absence of high pressures [18]. Adsorbed salivary conditioning films in the oral cavity are required to provide lubrication between the tongue and lingual tooth surfaces to facilitate speech, which occurs under low pressures of up to 150 kPa [19], but at the same time should withstand mastication forces on molar surfaces, which can yield pressures as high as 86 MPa [20], which is higher than the pressures occurring in articular joints [17].

Another unique feature of the oral cavity is that the adsorbed lubricating film is not solely perturbed by disease, but perturbations of its structure and composition occur several times per day during speaking, eating, drinking and tooth brushing. Recently, it has been demonstrated that the hydrophilicity of adsorbed salivary conditioning films in the oral cavity is preserved after chemical perturbation from oral health care products by a mechanism called “polar-apolar layering" [21], in which polar or a-polar salivary proteins are thought to adsorb in case a perturbation increases or decreases the hydrophilicity of the tooth surface, respectively. To this end, the oral cavity is equipped with different salivary glands (parotid, submandibular, sublingual, and other minor glands), all excreting different proteins. Mucinous glycoproteins in adsorbed lubricating films are currently considered to yield both significant structural strength to adsorbed films as well as the necessary hydrophilicity to preserve sufficient amounts of water in the interface [11], [18], [22]–[24], although the role of glycoproteins in specific, has only been speculated upon.

In this study, we provide a comprehensive analysis of biolubrication in the human oral cavity, encompassing lubricating properties of conditioning films adsorbed from saliva excreted by the various major salivary glands, perturbation of the lubricating properties of salivary conditioning films by detergents and the corresponding changes in *in vivo* lubrication determined through sensory perception of tooth surfaces in a group of volunteers, with the aim of establishing the role of structural properties and glycosylation of conditioning films in biolubrication, taking the oral cavity as a model system.

## Results

### Structure and lubricity of *ex vivo* adsorbed conditioning films from different salivary glands and volunteers

**Figure 1:**
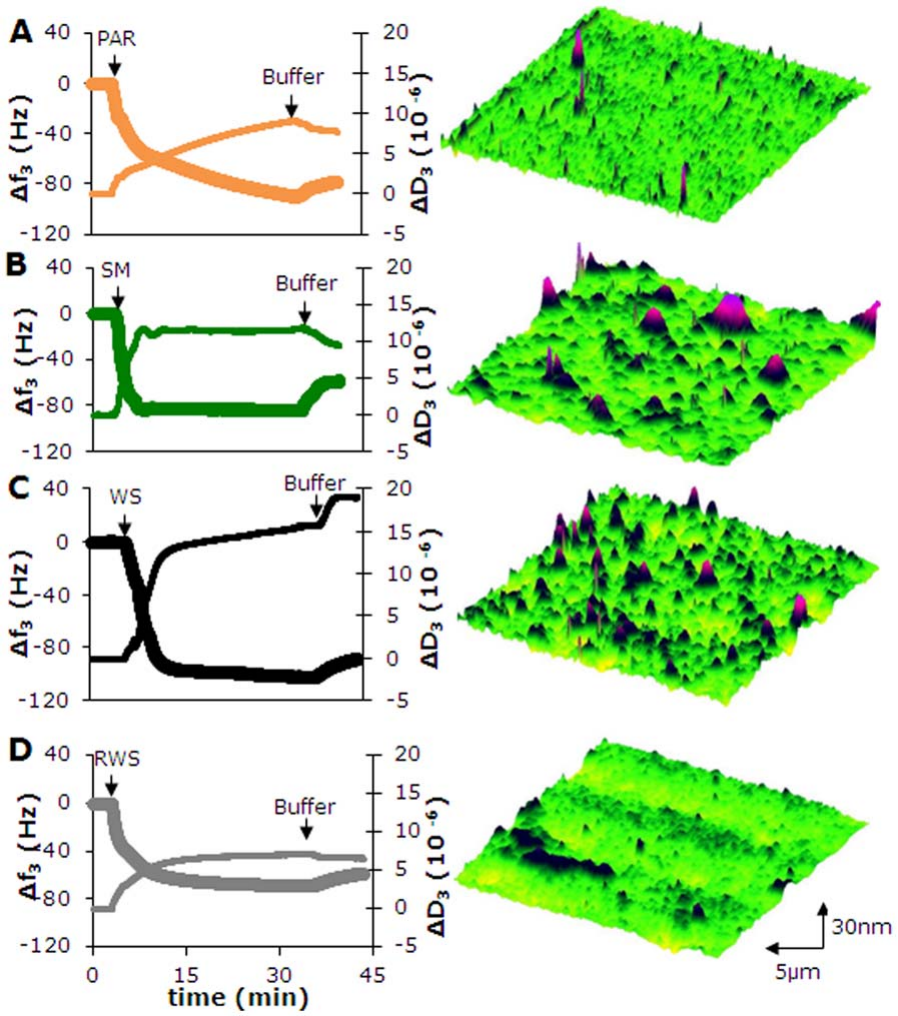
*Ex vivo* adsorption of proteins from salivas collected from different salivary glands. Example of the QCM-D response to salivary protein adsorption on hydroxyapatite crystals as a function of time, expressed in changes in third harmonic frequency (Δf3, thick line) and dissipation (ΔD3, thin line), together with AFM images of the topography of the adsorbed salivary conditioning films as observed at the end of an experiment. The QCM-D chamber is initially filled with buffer till a stable base-line is observed, after which saliva is introduced. After 30 min of salivary protein adsorption, the chamber is perfused again with buffer. (**A**) parotid saliva (PAR) from a single donor, (**B**) submandibular saliva (SM) from a single donor, (**C**) fresh whole saliva (WS) from a single donor, (**D**) reconstituted whole saliva (RWS) from a pool of donors.

**Figure 2:**
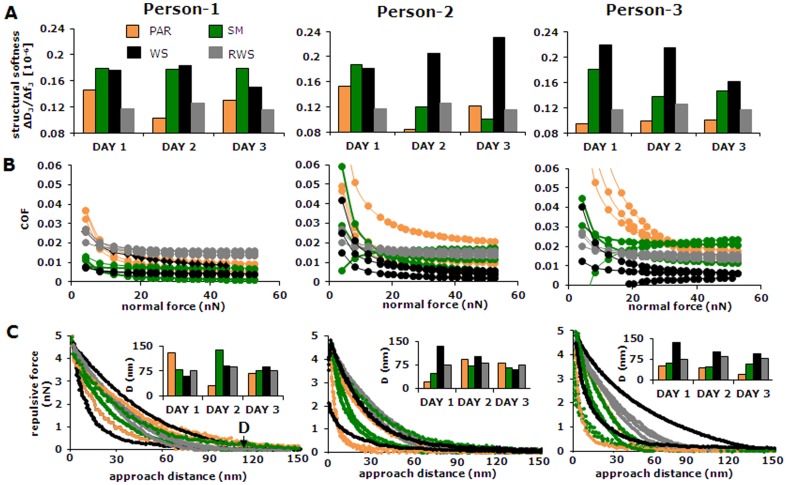
Structural softness, lubricity and repulsive forces of *ex vivo* adsorbed salivary films from different volunteers. (**A**) Day-to-day and person-to-person variations in the structural softness of 30 min old adsorbed films on hydroxyapatite-coated quartz crystals from parotid (PAR), submandibular (SM), fresh whole (WS) and reconstituted whole saliva from a pool of donors (RWS), taken as the ratio of dissipation (ΔD3) and frequency shift (Δf3) of the third harmonic resonance frequency of the QCM crystal. (**B**) The coefficient of friction (COF) as a function of the normal force applied between a colloidal probe and films formed from salivas collected from different salivary glands and volunteers (see also Figure S1). Data pertaining to salivas collected at different days are indicated by multiple lines, with colors corresponding with the labels in Figure 2A. (**C**) Repulsive force as a function of the approach distance between a colloidal probe and films formed from salivas collected from different glands and volunteers. Data pertaining to salivas collected at different days are indicated by multiple lines, with colors corresponding with the labels in Figure 2A. The distance D of the repulsive forces (inserts) is taken as the distance where the colloidal probe starts to experience a repulsive force >0.1 nN.

Differences in structural properties of adsorbed films from different salivary glands to hydroxyapatite and day-to-day and person-to-person variations were examined. Stimulated parotid, submandibular and fresh, whole saliva were separately collected from three volunteers (Figure S1). Reconstituted whole saliva was collected and pooled from a group of 20 volunteers. Subsequently, adsorption of the different salivas from individual volunteers and reconstituted whole saliva on hydroxyapatite was observed *in vitro* during 30 min using a quartz crystal microbalance with dissipation (QCM-D), after which the adsorbed films were imaged using colloidal probe atomic force microscopy (AFM) (Figure 1). QCM-D indicated that, regardless of its source, adsorbed saliva yields a decrease in resonance frequency and an increase in dissipation. Note that adsorption of submandibular saliva as well as of fresh, whole saliva yields larger globules of adsorbed proteins than does adsorption from parotid saliva and reconstituted whole saliva, as visualized by AFM. The visco-elasticity of these salivary films is indicated by the ratio (ΔD_3_/Δf_3_) and will be referred to in this manuscript as “structural softness" according to convention within the QCM community, as for instance by Höök *et al.* [25]. The structural softnesses of adsorbed conditioning films from the different salivas from individual volunteers and reconstituted whole saliva are summarized in Figure 2A. Major day-to-day and person-to-person variations are evident, while adsorption from reconstituted whole saliva yielded the least soft films with the smallest day-to-day variation. In general, adsorbed films from fresh whole saliva and submandibular saliva were softest. The softness of adsorbed films from parotid saliva was most variable across volunteers and from day-to-day. Comparable observations can be made with respect to day-to-day and person-to-person variations in the coefficient of friction (COF; Figure 2B), measured by lateral force colloidal probe AFM. Friction is generally low for films adsorbed from fresh, whole saliva and submandibular saliva, while highest on films from pooled, reconstituted whole saliva. However, these films showed the least day-to-day variations, while films from parotid saliva were most variable with respect to their COF across volunteers and from day-to-day. Note that the COF is initially high, but decreases with increasing loading force to stable values above a loading force of around 20 nN, regardless of the salivary source. The colloidal AFM tip experiences repulsive forces upon approach of the adsorbed films that depend on the salivary source and range up to 120 nm from the hydroxyapatite surface (Figure 2C). In general, the adsorbed films from parotid saliva are significantly (p<0.05, two tailed Student t-test) more variable from day-to-day and person-to-person than films adsorbed from submandibular saliva or fresh, whole saliva. Adsorbed films from reconstituted whole saliva show significant differences (p<0.05, two tailed Student t-test) in softness, coefficient of friction and repulsive force with respect to fresh, whole saliva but it clearly yields less variability between different experiments than the day-to-day and person-to-person variations between experiments with salivas from volunteers. Therefore, the remainder of this study was carried out with pooled, reconstituted whole saliva.

### Structure, composition and lubricity of perturbed salivary films *in vitro*

**Figure 3:**
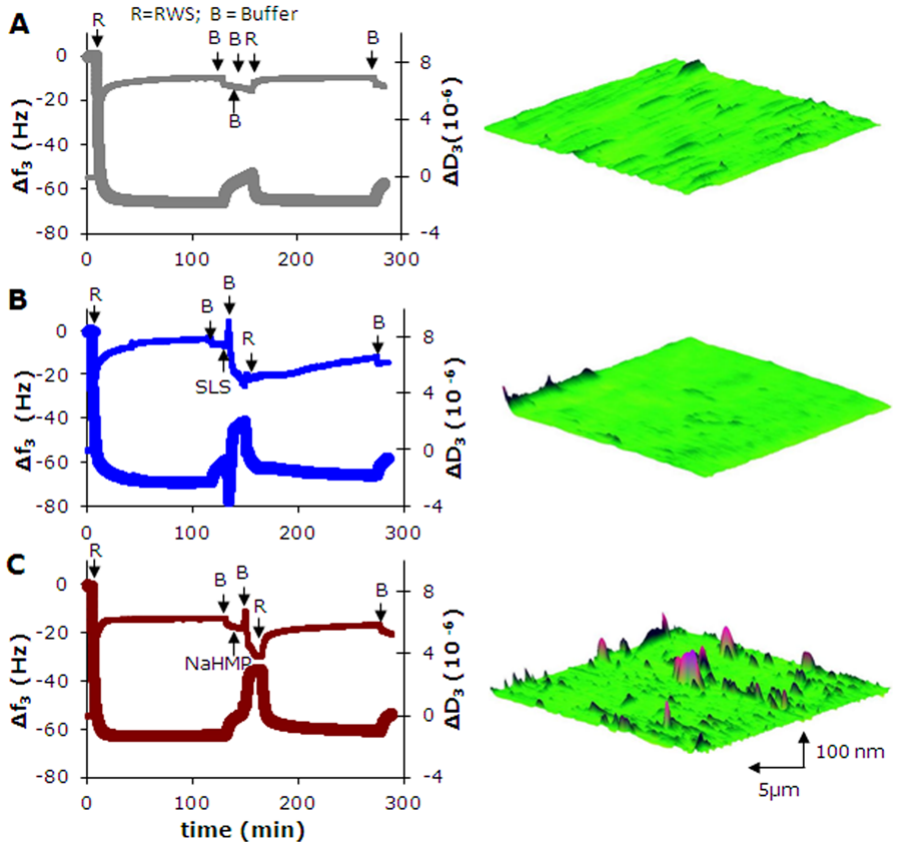
Influence of detergents on desorption and adsorption of salivary proteins. Example of the QCM-D response to protein adsorption from reconstituted whole saliva (RWS) on hydroxyapatite crystal surfaces, perturbation and continued adsorption of salivary proteins as a function of time, expressed in changes in third harmonic frequency (Δf3, thick line) and dissipation (ΔD3, thin line), together with AFM images of the topography of the adsorbed films as observed at the end of an experiment. Perturbation was established by exposure to (**A**) buffer, (**B**) SLS, (**C**) NaHMP. The QCM-D chamber is initially filled with buffer till a stable base-line is observed, after which RWS is introduced for 2 h to allow salivary protein adsorption, after which a buffer rinse is applied, followed by 2 min perturbation by buffer or a detergent, intermediate buffer rinsing for 15 min and continued perfusion of the chamber with RWS and at the end there was a final buffer rinsing, as indicated in the figure.

**Figure 4:**
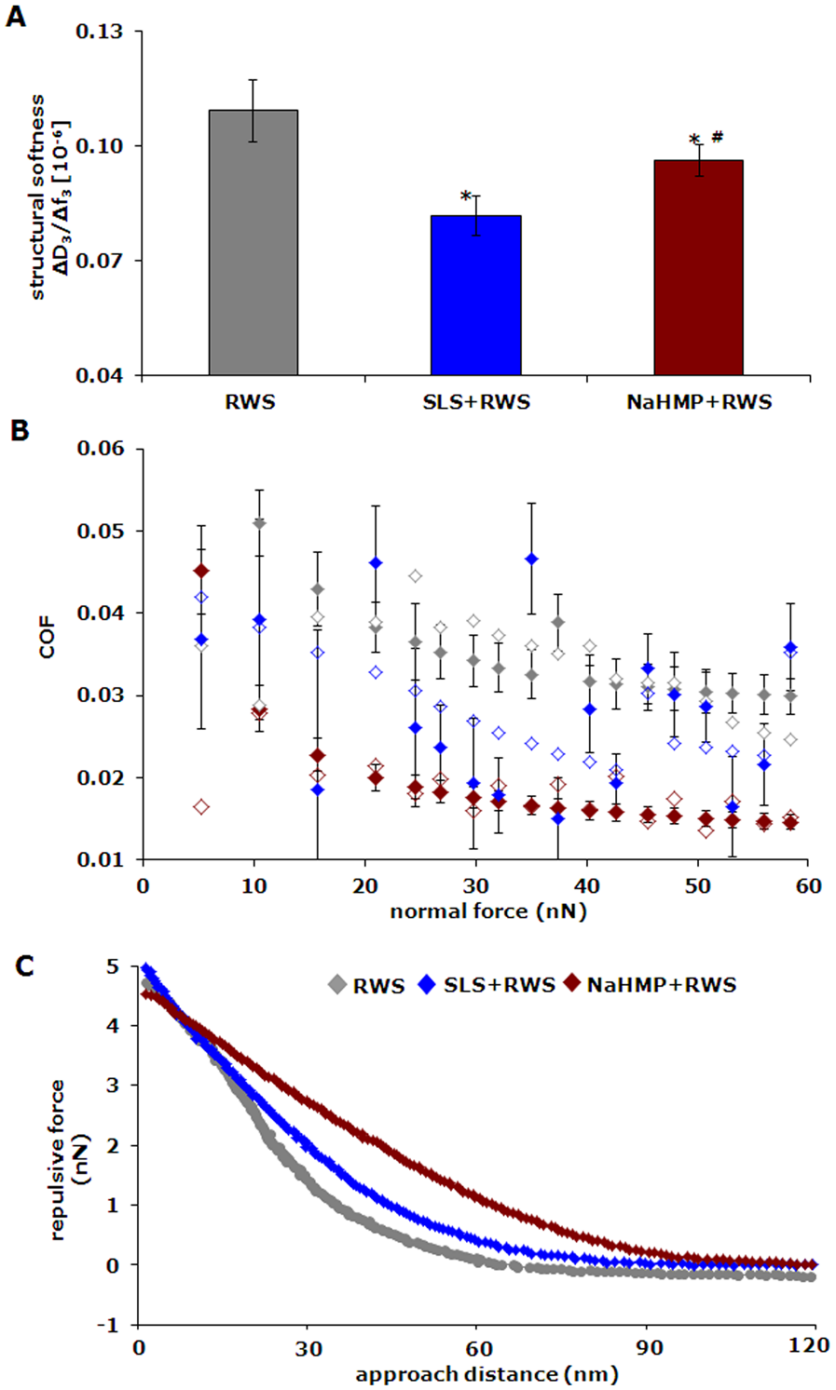
Structure and lubricity of the salivary conditioning films formed after perturbation by detergents. (**A**) Structural softness of salivary films adsorbed on conditioning films exposed to buffer (RWS), SLS (SLS+RWS) and NaHMP (NaHMP+RWS) and after final buffer rinsing, i.e. at the very end of an adsorption experiment (see also Figure 3). Error bars represent the standard deviations over five independent measurements of the structural softness. Statistically significant (p<0.05, two tailed Student t-test) differences in properties of the SLS+RWS and NaHMP+RWS films with respect to RWS are indicated by *signs. Differences in the properties of the NaHMP+RWS with respect to SLS+RWS is indicated by #sign. (**B**) Coefficient of friction as a function of the normal force applied for salivary films adsorbed on films exposed to buffer (RWS), SLS (SLS+RWS) and NaHMP (NaHMP+RWS). The COF during compression and de-compression of the films is represented by closed and open symbols, respectively. Error bars represent the standard deviations over 18 independent COF measurements. (**C**) Example of the repulsive force as a function of the approach distance for the different films.

Salivary films in the oral cavity are perturbed at least twice a day by detergents from toothpaste formulations, after which continued adsorption of salivary proteins occurs. This series of events was simulated in the QCM chamber by first adsorbing proteins from reconstituted whole saliva, followed by rinsing with buffer, sodium lauryl sulphate (SLS) or sodium hexametaphosphate (NaHMP), after which reconstituted whole saliva was perfused again through the chamber (Figure 3). Rinsing with buffer or detergent solutions was accompanied by increases in resonance frequency and decreases in dissipation, whereas continued adsorption of saliva yielded decreases and increases in resonance frequencies and dissipation, respectively. Note that continued salivary protein adsorption after rinsing with NaHMP stimulated the formation of large proteinaceous globules, not present after rinsing with SLS. Although the negative frequency shifts are comparable after continued salivary protein adsorption, a distinct influence of whether rinsing was done with buffer, SLS or NaHMP solutions becomes evident on the structural softness, coefficient of friction and the repulsive force experienced by the AFM colloidal probe (Figure 4). The structural softness of newly adsorbed films on top of conditioning films exposed to NaHMP or SLS is less compared with conditioning films formed on top of buffer exposed films (Figure 4A). At the same time, the coefficients of friction of adsorbed films after NaHMP exposure are lower than on films newly formed after buffer or SLS exposure (Figure 4B). Concurrently, films adsorbed on NaHMP exposed conditioning films repel the colloidal AFM tip strongest and over a considerably larger distance than observed for buffer or SLS (Figure 4C).

**Figure 5:**
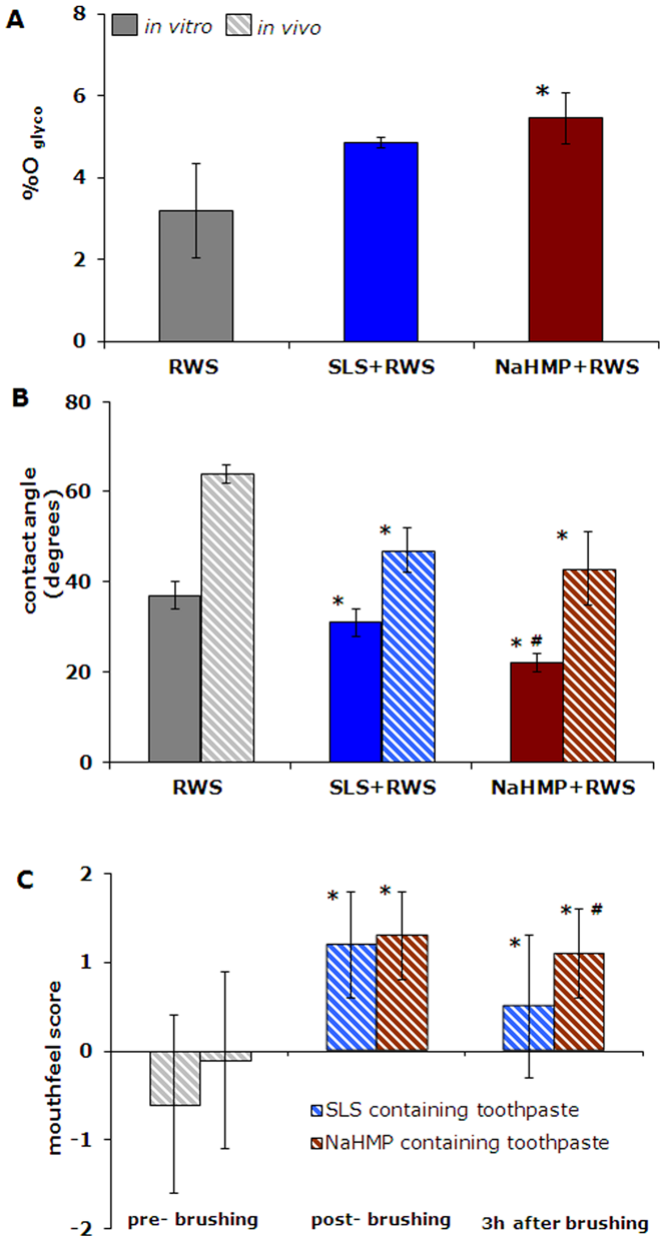
Glycosylation, clinically registered intra-oral contact angles and sensory perception of tooth surfaces *in vivo*. (**A**) The %O_glyco_ of salivary films adsorbed on conditioning films exposed to buffer followed by reconstituted whole saliva (RWS), SLS (SLS+RWS) and NaHMP (NaHMP+RWS). Error bars represent the standard deviations over three independent XPS measurements on differently prepared samples. Statistically significant (p<0.05, two tailed Student t-test) differences of the SLS+RWS and NaHMP+RWS films with respect to RWS films is indicated by *sign. (**B**) Water contact angles *in vitro* for the films described in Figure 5a and clinically registered, water contact angles measured on the front incisors of human volunteers prior to and after brushing with a SLS or NaHMP containing formulation (represented by hatched columns). Error bars represent the standard deviations over twelve independent contact angle measurements. For the *in vitro* contact angles, statistically significant (p<0.05, two tailed Student t-test) differences with respect to RWS films are indicated by *signs, while #sign indicates significant differences of NaHMP+RWS films compared with SLS+RWS ones. Similarly, for the *in vivo* contact angles *signs are used to show the significant difference with respect to unbrushed films and #sign indicates significant differences of brushed films with NaHMP toothpaste compared with brushed films with SLS toothpaste. (**C**) Mouthfeel scores prior to and after brushing with an SLS or NaHMP containing toothpaste formulation. The mouthfeel questionnaire involved the following questions: 1. How do you like the smoothness of your teeth? 2. How do you like the clean feeling of your teeth? 3. How do you like the moist feeling of your teeth? 4. Overall, how do you like the feeling of your mouth? Scoring was done on a five point scale according to: −2 = extremely bad smoothness, −1 = bad smoothness, 0 = neutral, 1 = good smoothness, and 2 = extremely good smoothness. Error bars represent the standard deviations over the scores obtained from 64 volunteers after use of an SLS containing toothpaste and 12 volunteers after use of a NaHMP containing toothpaste. Statistically significant (p<0.1) differences with respect to pre-brushing are indicated by *signs for each formulation, while #sign indicates significant differences between the SLS and NaHMP containing toothpaste.

X-ray photoelectron spectroscopy (XPS) was applied to determine the degree of glycosylation (%O_glyco_) of the different films *in vitro* (Figures S2 and Table S1). Glycosylation was higher after continued adsorption of salivary proteins on SLS or NaHMP exposed conditioning films, respectively (Figure 5A), which was accompanied by a decrease in water contact angles on the films (Figure 5B). Both the increase in the degree of glycosylation as well as the decrease in water contact angle was largest for continued adsorption after exposure to NaHMP.

### Clinically registered intra-oral contact angles and sensory perception of adsorbed films on teeth *in vivo*

Clinically registered water contact angles on the front incisors of human volunteers showed a significantly larger decrease after brushing with a NaHMP containing toothpaste formulations than after use of a SLS containing one (see also Figure 5B). This correspondence between *in vitro* and *in vivo* effects of both detergents stimulated us to investigate whether volunteers could sense these changes. To this end, volunteers were asked to score the smoothness of their teeth, as probed by the tongue, prior to and after brushing with a SLS or NaHMP containing toothpaste (Figure 5C). Mouthfeel improved significantly (p<0.1) to similar scores immediately after brushing with an SLS or NaHMP containing paste, and this effect lasted at least up to 3 h after brushing. However, volunteers continued to report a significantly smoother mouthfeel 3 h after use of the NaHMP containing formulation than after use of the SLS containing one.

## Discussion

Biolubrication is important throughout life, but its importance is felt more in its absence, as upon aging, disease or after injury. Currently, our understanding of biolubrication is insufficient to design effective therapeutics to restore biolubrication in the elderly and diseased. The oral cavity is an excellent model to study biolubrication as it constitutes a far more accessible site of the human body to derive lubricating fluid from than for instance the joint space or the vagina. Making use of the dynamics of the oral cavity with its daily perturbations due to tooth brushing and different glandular secretions, this study establishes for the first time the combined role of glycosylation and structure in biolubrication. *In vitro*, adsorbed salivary conditioning films were more lubricious when the structural softness and hydrophilicity increased. Importantly, it could be demonstrated that glycosylation contributed to the lubricating properties of the films. It is considered intriguing, that also *in vivo* hydrophilicity of tooth surfaces related with the smooth mouthfeel perception in a group of volunteers. Topographic images of salivary films revealed features (Figures 1 and 3) with a height of utmost 30–80 nm at an average roughness of 7±3 nm, unlikely to be probed by the tongue possessing roughness features itself with dimensions ranging up to tens of micrometers. Moreover, it has been demonstrated that the perception limit for roughness differences by the tongue is limited to 500 nm [26]. Thus it is concluded that the tongue senses changes in shear developing due to differences in the structure and glycosylation of the lubricating, adsorbed salivary film covering our teeth rather than roughness of the film.

With respect to biolubrication, both whole saliva as well as submandibular saliva yielded structurally softer and more lubricious adsorbed films than parotid saliva. Since the submandibular gland is known to excrete saliva that is more rich in glycosylated mucins [27], this is consistent with the role of glycosylation in establishing biolubrication. Reconstituted whole saliva from a pool of volunteers that had been freeze-dried and thawed showed the highest friction. Freeze-thawing does not alter the concentration of lower-molecular weight proteins in saliva which has been freeze-dried and stored at −20°C or −80°C for a period of 6 months [28]. However, based on the current results, an impact on the prevalence of high-molecular weight proteins by handling, including centrifugation, may not be ruled out [27], [28]. Nevertheless, reconstituted whole saliva from a pool of donors was preferred as it provides much more reproducible results than any of the glandular salivas, including whole saliva from an individual donor. Moreover, the coefficients of friction measured for salivary films adsorbed from reconstituted whole saliva correspond well with those measured *in vivo*, using an intra-oral device, attached to orthodontic brackets [29].

**Figure 6:**
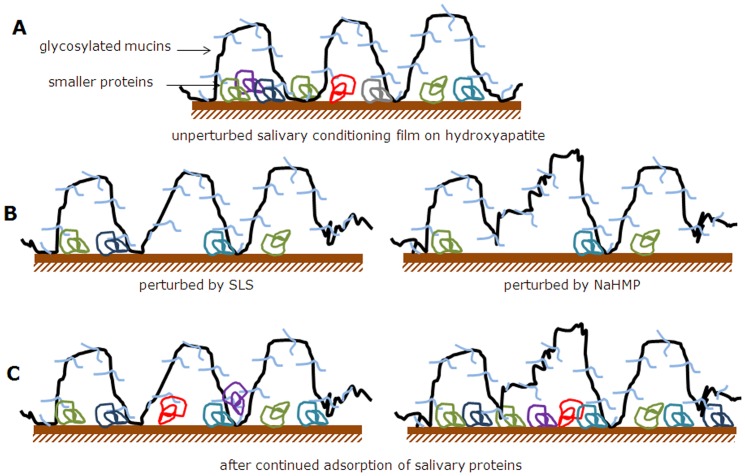
Schematic architecture of salivary conditioning films prior to and after chemical perturbation. (**A**) unperturbed salivary conditioning film, showing glycosylated mucins adsorbed in loops and trains over a layer of densely packed low-molecular weight proteins, including proline-rich proteins, histatins and lysozymes. (**B**) salivary conditioning film after exposure to detergents, showing removal of hydrophobic, smaller proteins and partial detachment of high molecular weight glycosylated mucins. Due to their larger size and multiple adsorption sites, larger proteins do not fully detach like the smaller ones. (**C**) salivary conditioning film after continued salivary flow over films exposed to detergents. Due to their smaller size, low molecular weight proteins adsorb faster than higher-molecular weight mucins, yielding a denser layer of adsorbed low-molecular weight proteins on the surface causing a greater structural softness and extended loops of glycosylated mucins, leaving the surface more hydrophilic. Smaller proteins have less chance to adsorb after exposure to SLS because more trains are left that occupy the substratum surface than after exposure to NaHMP.

It has been shown that on the mucosal surfaces of patients showing reduced salivary secretion, proteins like mucins and statherin were retained, but that without the presence of water functional integrity of these proteins is uncertain [30]. Water is needed for biolubrication, but needs a structural component in order to be maintained at an interface, especially when under pressure. The maximum normal force applied by the AFM in our studies corresponds with a mean pressure of 60 MPa and a maximal one of 90 MPa as calculated by a Hertzian model, which is in line with the maximum pressure of 86 MPa found on molar surfaces [20]. Our study shows that adsorbed glycosylated proteins are pivotal in providing biolubrication to adsorbed salivary films. Glycosylated proteins are known to bind water due to their hydrophilic properties and adsorbed to a surface will provide a scaffold that maintains water despite external pressures on the adsorbed film. Salivary glands secrete different types of low- and high-molecular weight proteins [27], [31] and according to the Vroman effect [32] describing protein adsorption from blood, these proteins will adsorb in a well defined sequence. First, small, low-molecular weight proteins will adsorb as their higher diffusional speed allows them to reach a surface faster than larger, high-molecular weight proteins. However, larger proteins have multiple binding sites with a surface and after their arrival at a surface will slowly displace the smaller, low-molecular weight proteins. A similar sequence of events has been suggested for oral salivary protein adsorption and secretory IgA and low-molecular weight proline-rich proteins were found to be displaced by higher-molecular weight proteins [33], [34]. Schematically, an adsorbed salivary protein film may look as presented in Figure 6A. Glycosylated, high-molecular weight mucins adsorb in loops and trains and provide a scaffold to contain and maintain water at the surface, while adsorbed smaller proteins like proline-rich proteins, histatins, lysozymes, amylases may be found underneath the loops and between the trains.

The adsorbed film as proposed in Figure 6A can be perturbed chemically and structurally by oral detergents. Although it is known that salivary films cannot be fully desorbed by tooth brushing and detergent action, it is unlikely that this statement relates to the smaller, low-molecular weight proteins and we envisage significant detachment of these proteins by detergents like SLS and NaHMP. Detergents are unlikely to desorb high-molecular weight proteins, but will probably break a number of bonds to cause more extensive looping of these adsorbed proteins (schematics in Figure 6B). SLS and NaHMP are both powerful detergents, but since NaHMP yields large scale reduction in the film mass, NaHMP is clearly the stronger detergent as confirmed by XPS analysis of dehydrated salivary film thickness [34]. NaHMP can thus cause greater detachment of trains, allowing more extensive looping of glycosylated proteins than does SLS, but not desorption of an entire molecule in line with the higher degree of glycosylation of these films found in XPS, close to the degree of glycosylation found for adsorbed films of pig gastric mucin only (see footnote to Table S1). Continued exposure of such films to saliva may allow adsorption of small proteins in between high-molecular weight trains that have remained on the surface. Loops with glycosylated groups will then extend for a considerable period of time into solution (Figure 6C), holding water molecules, providing long range repulsion towards the opposing surfaces and yielding low coefficient of friction *in vitro* and a smoother mouthfeel after use of a NaHMP containing toothpaste formulation *in vivo*. Eventually loops of high-molecular weight glycosylated proteins will displace adsorbed, smaller proteins and train-formation occurs again, eventually leading to surface thermodynamic homeostatis [21], although it has been demonstrated that adsorbed film perturbation by NaHMP *in vivo* may last minimally 24 h [35].

In summary, using a combination of QCM-D, AFM, XPS and water contact angle measurements *in vitro* and *in vivo*, we have demonstrated that biolubrication in the oral cavity is probably due to a combination of structure and the degree of glycosylation of adsorbed salivary protein films. Lubricating properties *in vitro* were confirmed by intra-oral, clinical contact angle measurements and mouthfeel evaluation *in vivo*. Therewith this is the first comprehensive study to demonstrate that biolubrication results from a combination of changes in structure and degree of glycosylation, relating smooth mouthfeel with lubrication at the molecular level. This may be an important clue to design effective therapeutics to restore biolubrication in the elderly and diseased. Most artificial salivas only contain mucins and the restoration of biolubrication achieved is highly temporary. Preparations containing low-molecular weight proteins as well may yield adsorbed films according to the architecture Figure 6C that are more stable and yield long-term relief of insufficient biolubrication.

## Materials and Methods

### Saliva collection

Human whole saliva from twenty healthy volunteers (10 male and 10 female subjects, average age 30±8 years) was collected into ice-chilled cups after stimulation of salivary flow by chewing Parafilm® according to the draining/spitting method described by Navazesh and Christensen [36]. The medical ethical committee approved collection of human saliva (approval no. M09.069162 and UMCG IRB #2008109) and patients gave their informed consent. After the saliva was pooled and centrifuged at 12,000 g for 15 min at 4°C, phenylmethylsulfonylfluoride was added to a final concentration of 1 mM as a protease inhibitor. The solution was again centrifuged, dialyzed for 24 h at 4°C against demineralized water, and freeze dried for storage at −20°C in order to provide for a stock. Finally, a lyophilized stock was prepared by mixing freeze dried material originating from a total of 2 l of saliva. Note that recently it has been shown that freeze-thawing does not alter saliva which has been stored on ice, at −20°C or −80°C for a period of 6 months [28]. Reconstituted, human whole saliva (RWS) was prepared from the lyophilized stock by dissolution of 1.5 mg/ml in buffer (2 mM potassium phosphate, 1 mM CaCl_2_, 50 mM KCl, pH 6.8) for experiments.

Parotid saliva was collected by citric acid stimulation and submandibular saliva was collected without any stimulation in ice-cooled beakers from three healthy volunteers (average age of 29±3 years). These saliva's were collected by applying an intra-oral device (Figure S1) suitable to separately collect parotid, and submandibular saliva (for details see Veerman *et al.* [37]). All saliva's were collected in the morning and used directly after collection in QCM-D experiments without any further interference. The collection of whole saliva, submandibular and parotid saliva from each volunteer was repeated on three different days.

### Quartz crystal microbalance with dissipation

The visco-elasticity or structural softness and formation kinetics of adsorbed salivary films was studied using a QCM-D device, model Q-sense E4 (Q-sense, Gothenburg, Sweden). Hydroxyapatite-coated quartz crystals, with a sensitivity constant of 17.7 ng/cm^2^ for a 5 MHz sensor-crystal, were used as substrata. Before each experiment, the hydroxyapatite coated crystals were rinsed in ethanol (100%) for 15 min, followed by drying with N_2_ and an UV/ozone treatment. At the start of each experiment, the sensor-crystal was incubated in buffer under flow. When stable base lines for the shifts in resonance frequency, Δf_3_ and dissipation, ΔD_3_ at third harmonics were achieved, saliva from the ice-cooled beaker collected directly from the intra-oral devices was introduced in the system by perfusion from the inlet to the outlet reservoir by a peristaltic pump (Ismatec SA, Glattbrugg, Switzerland). All salivas were perfused through the QCM-D chamber at 25°C for 30 min with a shear rate of approximately 3 s^−1^ followed by 15 min buffer rinsing to remove unbound proteins. This represents a low oral salivary flow rate as determined by Watanabe *et al.* [38]. Frequency and dissipation were measured real-time during perfusion.

For reconstituted whole saliva additional experiments were done, and adsorption was also monitored using QCM-D for 2 h, after which the chamber was perfused with SLS (2500 ppm) or NaHMP (2500 ppm) solutions or buffer for 2 min, followed by another 2 h of salivary flow to form a new film on top of the detergent-exposed one. In between each step as well as at the end of an experiment in preparation for other experiments, the chamber was perfused with buffer for 15 min.

After these experiments, hydroxyapatite crystals were removed from the QCM-D device and kept hydrated for immediate use in further experiments (see below).

### Colloidal probe atomic force microscopy

Coefficient of friction, surface topography and repulsive force range toward a colloidal AFM probe [39] were measured with an AFM (Nanoscope IV Dimension^tm^ 3100) equipped with a Dimension Hybrid XYZ SPM scanner head (Veeco, New York, USA) on the different adsorbed salivary conditioning films. To this end, rectangular, tipless cantilevers (length (*l*), width (*w*) and thickness (*t*) of 300, 35 and 1 µm, respectively) were calibrated for their exact torsional and normal stiffness using AFM Tune IT v2.5 software [40]. The normal stiffness (*K_n_*) was in the range of 0.01 to 0.04 N/m, while the torsional stiffness (*K_t_*) was in the range of 2 to 4×10^−9^ N m/rad.

Subsequently, a silica particle of 4.74 µm diameter (*d*) (Bangs laboratories, Fishers, IN, USA) was glued to a cantilever with an epoxy glue (Pattex, Brussels, Belgium) using a micromanipulator (Narishige group, Tokyo, Japan) to prepare a colloidal probe. The deflection sensitivity (*α*) of the colloidal probe was recorded on bare hydroxyapatite in buffer to calculate the applied normal force (*F_n_*) using
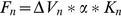
(1)where Δ*V_n_* is the voltage output from the AFM photodiode due to normal deflection of the colloidal probe.

The torsional stiffness and geometrical parameters of the colloidal probe were used to calculate the friction force (*F_f_*) [41], [42] according to
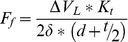
(2)where *t* is the thickness of the cantilever, *δ* is the torsional detector sensitivity of the AFM and Δ*V_L_* corresponds to the voltage output from the AFM photodiode due to lateral deflection of the colloidal probe. Lateral deflection was observed at a scanning angle of 90 degrees over a distance of 5 µm and a scanning frequency of 1 Hz. The scanning angle, distance and frequency were kept constant throughout all friction force measurements.

The colloidal probe was incrementally loaded and unloaded up to a maximal normal force of 60 nN in buffer. At each normal force, 10 friction loops were recorded to yield the average friction force. The coefficient of friction was measured by dividing the friction force with the respective normal force.

Repulsive force-distance curves between a colloidal probe and the films were obtained at a trigger threshold force of 5 nN and at a velocity of 10 µm/s. Repulsive force range D between the colloidal probe and the film was determined after correcting with the force range between the same colloidal probe and the hard, uncoated hydroxyapatite surface [43], for 40 repulsive force-distance curves. All the surface topography imaging by colloidal probe was performed at 1 nN of normal force.

### X-ray photoelectron spectroscopy

The degree of glycosylation of the adsorbed salivary films was determined by using XPS (S-probe, Surface Science Instruments, Mountain View, CA, USA). First, films adsorbed on hydroxyapatite-coated quartz crystals as removed from the QCM-D chamber, were dried in the pre-vacuum chamber of the XPS, and then subjected to a vacuum of 10^−7^ Pa. X-rays (10 kV, 22 mA), at a spot size of 250×1000 µm, were produced using an aluminum anode. Scans of the overall spectrum in the binding energy range of 1–1100 eV were made at low resolution (pass energy 150 eV). The area under each peak was used to yield elemental surface concentrations for C, O, N, Ti and Ca after correction with sensitivity factors provided by the manufacturer. The O_1S_ peak was split into three components, i.e. for oxygen involved in amide groups (C = O-N; 531.3 eV), carboxyl groups (C-O-H; 532.7 eV) and oxygen arising from the hydroxyapatite crystal. Accordingly, the fraction of the O_1s_ peak at 532.7 eV (%O_532.7_) was used to calculate the amount of oxygen involved in glycosylated moieties (%O_glyco_)
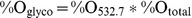
(3)where %O_total_ is the total percentage of oxygen.

### Contact angle measurements *in vitro*

Hydroxyapatite-coated quartz crystals with adsorbed protein films were allowed to air dry 45 min in order to obtain stable, so-called “plateau" water contact angles [44] of the advancing type, as measured by the sessile drop technique using a home-made contour monitor.

### Intra-oral contact angle measurements and mouthfeel evaluation

Ten volunteers were provided with a tube of a SLS (Crest regular®, Proctor and Gamble, Ohio, USA) or NaHMP (Crest vivid white night®, Procter and Gamble, Ohio, USA) containing toothpaste, along with an Oral B 40 (Oral B 40 Regular Toothbrush, Oral-B Laboratories Inc., California, USA) tooth brush. Volunteers were instructed to brush their teeth twice a day according to their habitual routine with the assigned toothpaste and not to use any other oral health care products. During the subsequent week, volunteers visited the dental clinic on three separate days for intra-oral water contact angle measurements at three times each day: pre-brushing in the morning, post-brushing (immediately after brushing) in the morning, and 3 h after brushing (pre-lunch). For morning evaluations, volunteers reported to the clinic prior to morning tooth brushing and before breakfast, eating or drinking.

Water contact angles were measured on the front incisors of the volunteers employing the sessile drop technique [45]. Small water droplets (1–2 µl) were placed on the tooth surface and a color slide was taken, from which the height and base-width of the droplets were measured and the contact angle was calculated. After water contact angles were taken, volunteers brushed with their assigned paste for one minute and thoroughly rinsed their mouth with tap water and water contact angles were measured again, as described above. Volunteers reported back after 3 h prior to lunch, allowing at least 1 h since eating and drinking.

Prior to contact angle measurements, volunteers filled out a questionnaire (Figure 5C) to score their mouthfeel.

### Statistical Analysis

The properties of the films adsorbed from the different salivas were pair-wise compared using a two tailed Student t-test. Mouthfeel scores were treated as continuous response variables and analysed using a linear mixed-effects model to adjust for the fact that repeated responses from a given volunteer may be correlated. Significance was established using the ANOVA F-test and Tukey's pair-wise comparison.

## Supporting Information

Figure S1 Procedure for collecting salivas from volunteers. (**A**) The Lashley cup for collecting parotid saliva. The Lashley cup (see insert) consists of an inner and outer chamber. The inner chamber is used for collecting saliva, while a slight underpressure is put on the outer chamber to stick the cup to the oral mucosa. The Lashley cup is placed over the orifice of the parotid duct. In the tube connected with the inner chamber flow of parotid saliva is clearly visible. In our study, parotid saliva was collected simultaneously from the right and left parotid gland under citric acid stimulation. The parotid saliva was collected into an ice-cooled beaker. (**B**) The segregator (see insert) for collecting the submandibular and sublingual saliva from the floor of the mouth. The central chamber of the segregator covers the orifices of the submandibular duct (Wharton's duct), while the lateral chambers cover the orifices of the sublingual ducts (ducts of Rivinus) that drain directly into the floor of the oral cavity. Besides the Rivinii ducts, the sublingual gland has also a Bartholin's duct that drains via the same orifice as the Warthon duct, due to which the submandibular saliva is contaminated with some sublingual saliva. As the flow rate of sublingual saliva is very low, this contribution will be minor. With regard to the very viscous sublingual saliva, a far too low volume of saliva was collected via the lateral chambers to allow for performing the experiments described in this paper. Submandibular saliva was collected into an ice-cooled beaker in absence of external stimulation. (**C**) Whole saliva was stimulated by chewing Parafilm® and collected into an ice-cooled beaker. The minimum amount of whole and glandular saliva needed for the various experiments was 2 ml per person.
(TIF)

Figure S2 O_1s_ photo-electron peak for salivary conditioning films formed after perturbation by detergents. (**A**) unperturbed salivary conditioning film from saliva (RWS) after exposure to buffer only. (**B**) salivary conditioning film after exposure to SLS and continued saliva (RWS) flow. (**C**) salivary conditioning film after exposure to NaHMP and continued saliva (RWS) flow. The O_1s_ photo-electron peak is decomposed in three components, due to oxygen involved in amide groups (C = O-N; binding energy 531.3 eV), carboxyl groups (C-O-H; 532.7 eV) and oxygen involved in other chemical functionalities in the substratum.
(TIF)

Table S1 Elemental surface compositions of adsorbed salivary conditioning films from different sources on hydroxyapatite crystal surfaces, together with the %O_glyco_. Data for stimulated parotid (PAR), submandibular (SM) and whole saliva (WS) are given separately for each volunteer.
(TIF)
