# Supplementary figures and images for: Role of Structure and Glycosylation of Adsorbed Protein Films in Biolubrication
Source: PLoS One. 2012 Aug 15;7(8):e42600. doi: 10.1371/journal.pone.0042600 (PMC3419733; doi:10.1371/journal.pone.0042600)

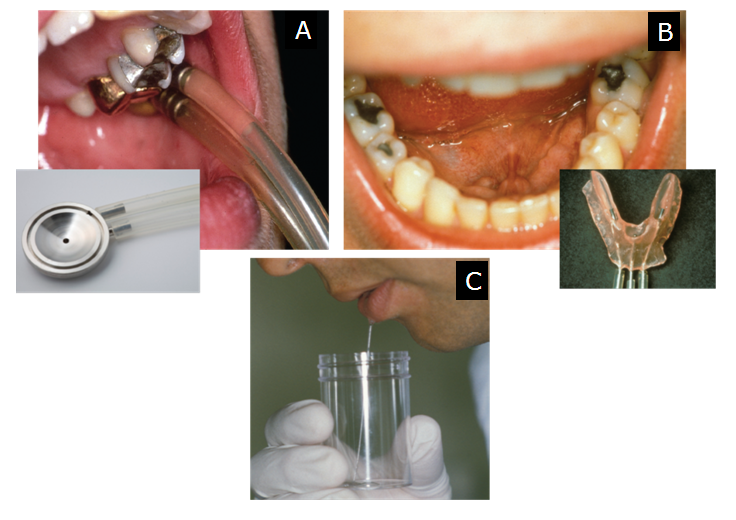

Supplement: Figure S1 — Procedure for collecting salivas from volunteers. (A) The Lashley cup for collecting parotid saliva. The Lashley cup (see insert) consists of an inner and outer chamber. The inner chamber is used for collecting saliva, while a slight underpressure is put on the outer chamber to stick the cup to the oral mucosa. The Lashley cup is placed over the orifice of the parotid duct. In the tube connected with the inner chamber flow of parotid saliva is clearly visible. In our study, parotid saliva was collected simultaneously from the right and left parotid gland under citric acid stimulation. The parotid saliva was collected into an ice-cooled beaker. (B) The segregator (see insert) for collecting the submandibular and sublingual saliva from the floor of the mouth. The central chamber of the segregator covers the orifices of the submandibular duct (Wharton's duct), while the lateral chambers cover the orifices of the sublingual ducts (ducts of Rivinus) that drain directly into the floor of the oral cavity. Besides the Rivinii ducts, the sublingual gland has also a Bartholin's duct that drains via the same orifice as the Warthon duct, due to which the submandibular saliva is contaminated with some sublingual saliva. As the flow rate of sublingual saliva is very low, this contribution will be minor. With regard to the very viscous sublingual saliva, a far too low volume of saliva was collected via the lateral chambers to allow for performing the experiments described in this paper. Submandibular saliva was collected into an ice-cooled beaker in absence of external stimulation. (C) Whole saliva was stimulated by chewing Parafilm® and collected into an ice-cooled beaker. The minimum amount of whole and glandular saliva needed for the various experiments was 2 ml per person. (TIF) [file pone.0042600.s001.tif]

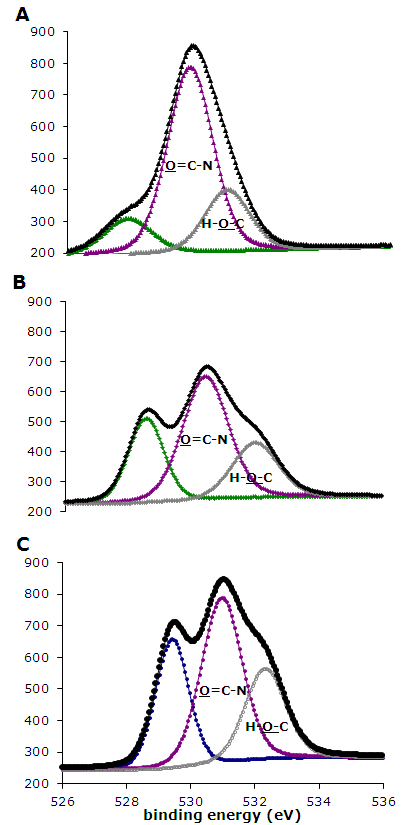

Supplement: Figure S2 — O1s photo-electron peak for salivary conditioning films formed after perturbation by detergents. (A) unperturbed salivary conditioning film from saliva (RWS) after exposure to buffer only. (B) salivary conditioning film after exposure to SLS and continued saliva (RWS) flow. (C) salivary conditioning film after exposure to NaHMP and continued saliva (RWS) flow. The O1s photo-electron peak is decomposed in three components, due to oxygen involved in amide groups (C = O-N; binding energy 531.3 eV), carboxyl groups (C-O-H; 532.7 eV) and oxygen involved in other chemical functionalities in the substratum. (TIF) [file pone.0042600.s002.tif]

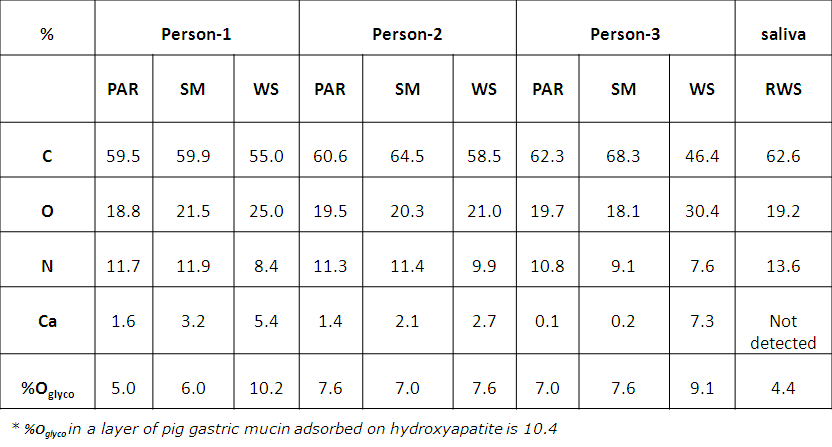

Supplement: Table S1 — Elemental surface compositions of adsorbed salivary conditioning films from different sources on hydroxyapatite crystal surfaces, together with the %Oglyco. Data for stimulated parotid (PAR), submandibular (SM) and whole saliva (WS) are given separately for each volunteer. (TIF) [file pone.0042600.s003.tif]
